# Sensing beyond Senses: An Overview of Outstanding Strides in Architecting Nanopolymer-Enabled Sensors for Biomedical Applications

**DOI:** 10.3390/polym14030601

**Published:** 2022-02-03

**Authors:** S. Malini, Arpita Roy, Kalyan Raj, K. S. Anantha Raju, Ismat H. Ali, B. Mahesh, Krishna Kumar Yadav, Saiful Islam, Byong-Hun Jeon, Sean Seungwon Lee

**Affiliations:** 1 Department of Chemistry, B.M.S. College of Engineering, Bangalore 560019, India; kr.chem@bmsce.ac.in; 2 Department of Biotechnology, School of Engineering & Technology, Sharda University, Greater Noida 201310, India; arbt2014@gmail.com; 3 Department of Chemistry, Dayananda Sagar College of Engineering, Bangalore 560078, India; iamananthkurupalya@gmail.com; 4 Department of Chemistry, College of Science, King Khalid University, P.O. Box 9004, Abha 61413, Saudi Arabia; ihali@kku.edu.sa; 5 Department of Chemistry, JSS Academy of Technical Education, Bangalore 560060, India; bmahesh@jssateb.ac.in; 6 Faculty of Science and Technology, Madhyanchal Professional University, Ratibad, Bhopal 462044, India; envirokrishna@gmail.com; 7 Civil Engineering Department, College of Engineering, King Khalid University, Abha 61421, Saudi Arabia; sfakrul@kku.edu.sa; 8 Department of Earth Resources & Environmental Engineering, Hanyang University, 222-Wangsimni-ro, Seongdong-gu, Seoul 04763, Korea; bhjeon@hanyang.ac.kr

**Keywords:** nanosensors, nano-enabled, multifunctional nanomaterial, transduction, nano detection, nano quantification

## Abstract

Nano-enabled sensing is an expanding interdisciplinary field of emerging science with dynamic multifunctional detecting capabilities, equipped with a wide range of multi-faceted nanomaterial having diverse dimensions and composition. They have proven to be highly robust, sensitive, and useful diagnostic tools ranging from advanced industrial processes to ordinary consumer products. As no single nanomaterial has proved to be unparalleled, recent years has witnessed a large number of nanomaterial-based sensing strategies for rapid detection and quantification of processes and substances with a high degree of reliability. Nano-furnished platforms, because of easy fabrication methods and chemical versatility, can serve as ideal sensing means through different transduction mechanisms. This article, through a unified experimental-theoretical approach, uses literature of recent years to introduce, evaluate, and analyze significant developments in the area of nanotechnology-aided sensors incorporating the various classes of nanomaterial. Addressing the broad interests, the work also summarizes the sensing mechanisms using schematic illustrations, attempts to integrate the performance of different categories of nanomaterials in the design of sensors, knowledge gaps, regulatory aspects, future research directions, and challenges of implementing such techniques in standalone devices. In view of a dependency of analysis and testing on sustained growth of sensor-supported platforms, this article inspires the scientific community for more attention in this field.

## 1. Introduction

The last decade has witnessed extensive research in sensing technology, largely attributed to the rapidly growing development of nanomaterial. As detection technologies adapting nanotechnology allows for the construction of smaller, sensitive, reliable, and, in some cases, self-powered sensors, many efforts by the researchers are under way to improvise their applications to solve various current domestic and industrial problems. Similarly, engineering novel materials with the aid of other technical disciplines to meet the sensing requirements would widen the possibility of developing smart structures with high sensing efficiency. However, the properties of an “ideal” sensor material largely depend on the context of an application addressed, and conducting frontier research along with a discussion of the current trends in this area becomes highly crucial to reach a broad set of inter-disciplinary applications.

A sensor may be described as a real time information acquisition device whose activity is based on interaction of the physical or chemical part of the sensor with some external analyte species resulting in a signal at different sensing situations. In accordance with the International Union of Pure and Applied Chemistry, a chemical sensor is a tool that interchanges chemical information arising from the properties of the analyte into an analytically useful signal. This interaction, often referred to as ‘transduction’, becomes more effective, rugged, reliable, and sensitive when enabled by nanomaterials with sensing properties. The enhanced sensitivity of the nanomaterial transducer is due to its small size, which helps identify specific molecules, the high surface area to volume ratio that helps to pack many active areas in small area, and the functionalization, which allows the tracking of multiple analytes.

The emerging nanotechnology enabled sensors permeate the diverse application domains of scalable manufacturing [1] and the transformation of sociotechnical scenarios with the world market projections predicted to exceed USD 10 billion, according to analysts of the global industry.

A case study report by Di Lecce et al. [2] presents a thorough picture with a focus on the market towards smart sensors that interact with other systems. The authors optimistically report the design and sensor implementations that encompass the emergence of microcontroller devices with sensory processing that undergo a change from mere measurement to incorporating artificial intelligence. Looking briefly at the economic scenario of sensors, one can witness a significant rise in market value reaching one billion units per every year with a modest rise in computing speed, mobile communication, and early intervention and monitoring of chronic diseases.

In anticipation of this enormous economic impact of nanomaterials, former President Clinton founded the National Nanotechnology Initiative (NNI) in the year 2000. This establishment with an initial investment of $1 billion has gone a long way in providing a multi-agency umbrella for the implementation of nanoscale devices with a budget request inflating up to $2.7 billion in 2020. Additionally, with federal agencies attracting government investments in nano-enabled devices, explosive growth is anticipated by 2020–2022 with the use of more than a trillion sensors [3] with advances in wireless sensor technology. While the forces to innovate are strong, the prime impetus for miniaturization comes from the electronics industry, water decontamination bodies, pharmaceutical researchers, and healthcare organizations, to develop nanosensing tools which are smaller and, therefore, faster. However, a sustained growth over the next decade is only possible with the involvement of sensor-supported functionalities from conceptual stage to commercial implantation.

The primary concern relating to efficient usage of nano-enabled sensing is the surface charge characteristics of nanomaterial that influences the subsystems and biological membranes. Reports by Martin K. Rasmussen et al. [4] show pioneering results on charge characterization applied to exosome-based diagnostics and Lingyun Xie et al. [5], who explored the solid-state reference electrode paired with InN/InGaN QD sensing electrode generating super-Nernstian response in electrochemical sensors confirmed by Kelvin probe force microscopy, which also emphasizes the spatial surface charge engineering and the influence of charge distribution at the nanoscale sensing. The ever-growing market of nanosensors can become sustainable by a combination of competitive and complimenting development of microscopic nanomaterial research and macroscopic manufacturing techniques.

Despite numerous reviews on nano-adapting sensors published in the past, the area of nano-sensing technology is extremely broad and, hence, requires an updated overview, such as the current one, which helps us appreciate the omnipresence of sensors, identify the latest sensing materials available, understand its vivid applications, decipher the underlying working mechanism, and motivate further development in this field. The structure of this review envisions and attempts to focus on the latest developments, innovations, safety concerns, discuss safety issues, unresolved problems, economic and regulatory aspects, along with sensing mechanisms encountered in the field of architecting nanomaterial-enabled sensors.

## 2. Properties of Nanomaterial in Sensors

**Figure 1:**
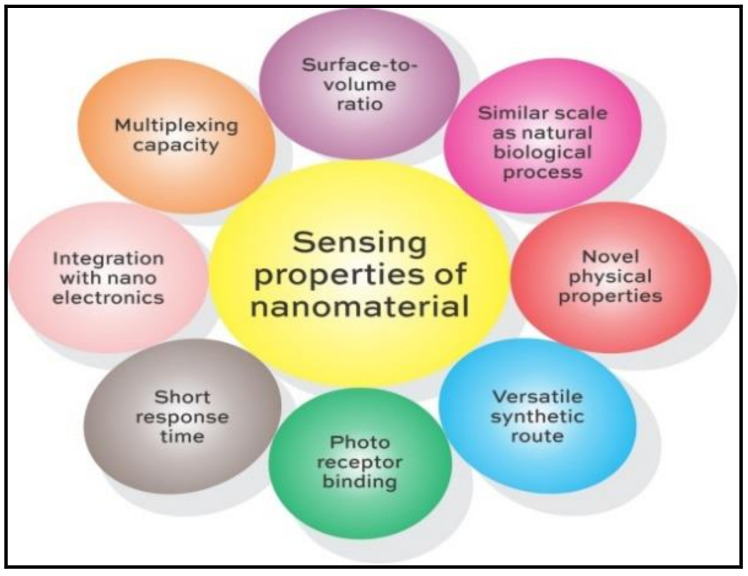
Relevant properties of nanomaterial in view of sensing ability.

The unique properties of nanoprobes, as represented in Figure 1, have tremendously contributed in raising the specificity of entrapping the analyte molecules. The ability of a sensor is largely decided by its capacity of resolving and processing a signal, which in turn depends on the constituting material.

Nanomaterial used as sensors must be sensitive, selective, and stable and are often designed using interfacing scheme with amplification strategy [6] and electrostatic induction [7]. Properties of nanomaterial, such as triggering redox reaction towards H_2_S by lattice oxygen [8], generating invisible near-infrared patterns through photoreceptor-binding to retina [9], charge transfer phenomenon through altering hopping distance of the charge carrier exhibited in the composites of polyindole/MoO_3_-WO_3_ [10], and surface reactions at Mesoporous silicon layers [11] sensitive to CO_2_, are thoroughly explored sensor materials.

Generally, an amplification of signal response is observed by reducing the order of dimensionality of the sensing material and, hence, a sequential integration of low dimensional materials is expected to have high sensing capabilities. Despite the problems of integration in zero-dimensional nanomaterials, unique zero-dimensional electronic carbon quantum dot structures, which enable detection at the molecular level [12], and one-dimensional nanomaterial [13] are well known for their large surface-to-volume ratio, high sensitiveness, and compactness, and exhibit enhanced sensing performance with a quick response. However, two dimensional nanomaterials, when compared to 0D, 1D, and 3D counterparts, are more preferable while devising a compact sensor due to the ease with which they form electrical contacts, a larger lateral size and diversity in configuration, flexibility in morphological properties, and their compatibility with thin films. Accordingly, a novel platform for multiplexed in vitro recognition of DNA in a virus causing hepatitis A & B through tracking of dispersal of K+ ions, reported by Yuanyuan Tian et al. [14], and 2D nanomaterial synthesized by thermal assistance [15] capable of resolving the issues associated with steady-state signals in gas sensors, emphasizes the significance of 2D nanomaterial.

Furthermore, an understanding of these remarkable physical, thermal, optical, and chemical properties in relation to the structure is facilitated by notable reviews in the past decade, listed in Table 1. Numerous sensing techniques, such as Raman scattering aiding the detection of therapeutic drug at nano-molar concentrations [16], micro-plasma technology powering UV sensing of ZnO nanowire [17], fluorescence technique [18] for lifetime imaging, and chemiluminescence resonance energy platform [19], are a subject of many publications allowing the investigation of structural, dimensional, and compositional features.

## 3. Transformational Applications of Nanomaterial for Sensing Phenomena

The advancements in nanotechnology have tremendously supported the principle need of extreme selectivity of sensing systems through highly efficient recognition, quantification, and signal processing. With the raising federal government annual funding for nanotechnology standing at over $1.6 billion today, the applications and benefits of nano-adapted sensors are playing a significant role in diverse areas. Despite the challenges of ensuring consistent reliable sensing, the strength of the nanotechnology-enabled sensing lies in the combatable binding chemistry with the sensing element at nanoscale. A glimpse into the recently realized applications is outlined under the following subheadings.

### 3.1. Agriculture

Application of nanomaterials in agriculture, in the form of nanopesticides and nanofertilizers, has raised the opportunities to minimize the use of toxic agrochemicals and enhance plant growth. As sustainable practices in farming are crucial to lessen the greenhouse emissions, benefiting the ecosystem and increasing the productivity of the land, nanotechnology holds huge promise for the agricultural sector. An active response of these materials towards promoting plant growth [20] and exhibiting salinity stress tolerance [21] shows new possibilities in this area of study. In this regard, nanostructured sensors can foster diverse approaches towards tailor-made irrigation practices through an analysis of wettability, pesticide residue, humidity, crop maturity, and yield mapping. Table 2 presents the functionality and consequences of different types of nanomaterials as sensors in agriculture.

Monitoring soil moisture is one of the major steps toward optimized irrigation, as realized through a highly consistent Graphene oxide array [22] sensor probe micro-fabricated with dimensions 22 × 4 × 0.5 cm^3^ implanted, with a sequence of five micro-sized sensors that could respond quickly to soil moisture in depth with a short recovery time. This ultra-thin probe functioning at a convenient temperature of 25 °C is inexpensive, consumes very low power, exhibits a deviation of only 4% and makes soil matrix profiling easier.

**Figure 2:**
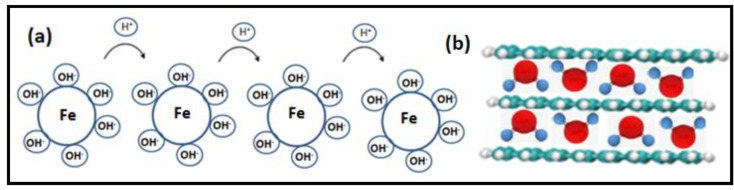
Sensing of soil moisture (**a**) chemical bond or (**b**) interchelation across layers.

The soil moisture and plant growth is appreciably affected by variation in atmospheric humidity and, hence, accurate measurement of humidity becomes crucial. An efficient humidity sensor [23] has recently been reported with cerium doped Mn-Bi ferrite nanoparticles. With an elevating humidity, the substance absorbs water on its surface forming Mn_0.95_Bi_0.05_Fe_2_–xCe_x_O_4_ with x varying from 0.005 to 0.03 and an electrostatic force develops over the sensing sites. In the presence of moisture, OH^−^ ions bond with Fe^3+^, while the H^+^ ions hop from one site to another causing a decrease in resistance, as shown in Figure 2a. An in-field integration of these sensors is achieved, engaging nano oxides of molybdenum and vanadium [24] using a capacitive sensor platform for evaluating silty and loamy soil. The sensing mechanism is reported to involve water molecules binding firmly across the interlayers of sensing material, depicted in Figure 2b, as comparable to grapheme oxide or might occur via polarization of water adsorbed on the interface of sensor, which became evident through transient impedance analysis, which measured the elevation in capacitance during proton transfer between adjacent water molecules.

The identification of moisture in the soil having a direct correlation with humidity has also promoted the development of a humidity-sensing [25] platform of polymeric nanocomposite with carbon nanohorns. A similar synergistical application is a polymer based molecularly imprinted nanocomposite sensor implemented in detecting and monitoring the levels of Cypermethrin [26], a popularly used pesticide and polypyrrole incorporated nanographene nanocomposite, in the detection of a well-known herbicide named “Paraqut” [27]. These detectors are often evaluated for their linear response and accuracy using electroanalytical techniques, such as Chronoamperometry square wave and cyclic voltammetry, under optimized conditions. One of the research groups has also used analytical measurement of the fluorescent intensity of green carbon dots synthesized [28] to establish the linearity of the response with the concentration of compounds exhibiting insecticidal activity, such as Diazinon, pesticide glyphosate, and herbicide amicarbazone. Fluorescence detection technology, using carbon dots prepared by one-step hydrothermal treatment, has been highly valued in the scientific community and has been applied to the quantification of organophosphorus pesticides in fortified tomatoes by quantum dots of Cadmium Selenium coupled with Zinc Sulfide [29]. These developments in sensing methods, which directly use quantum dots devoid of molecular recognition elements, detects the pesticide residues that may harm living organisms even in very small quantities. Nevertheless, the remarkable benefits of farm nanosensors performing the tasks that farmers cannot might be overshadowed by their drawbacks, such as high cost and requirement of years of data. Furthermore, the release of free nanoparticles from these sensors is expected to give rise to significant environmental issues, such as accumulation in soil, reduced root firmness, and growth disorders in plants, which cannot be resolved easily, and, hence, the future developments in this area require a scientific evaluation.

### 3.2. Biological Detection

Nano-enabling has drastically transformed biological detection starting from cell imaging to real life applications, bridging nanoscience with biological processes. Biological sensing is broadly categorized into two kinds, one based on inherent cell response and the other based on the engineered sensing material, which is popularized due to its field-portability and versatility.

Tracking and quantifying biological molecules demands extremely sensitive, reliable platforms, which, at times, may not be catered for by conventional approaches. This has driven researchers to explore unique systems, such as combined carbon nanotubes of malt-extracted hydrogel [30] capable of simultaneously monitoring the various growth phases of microorganisms, graphene to recognize body fluids such as glutathione [31,32], and membranes inserted by nanorods [33] that aid in the detection of membrane potential. The latter setup of membrane sensing has been successfully translated to peptide-coated nanorods to detect the mean modulation response of a cell’s membrane through quantum confined Stark effect. Such an amalgamation of inorganic-biological nanomaterial system has also facilitated the development of sensors for bacterial detection.

**Figure 3:**
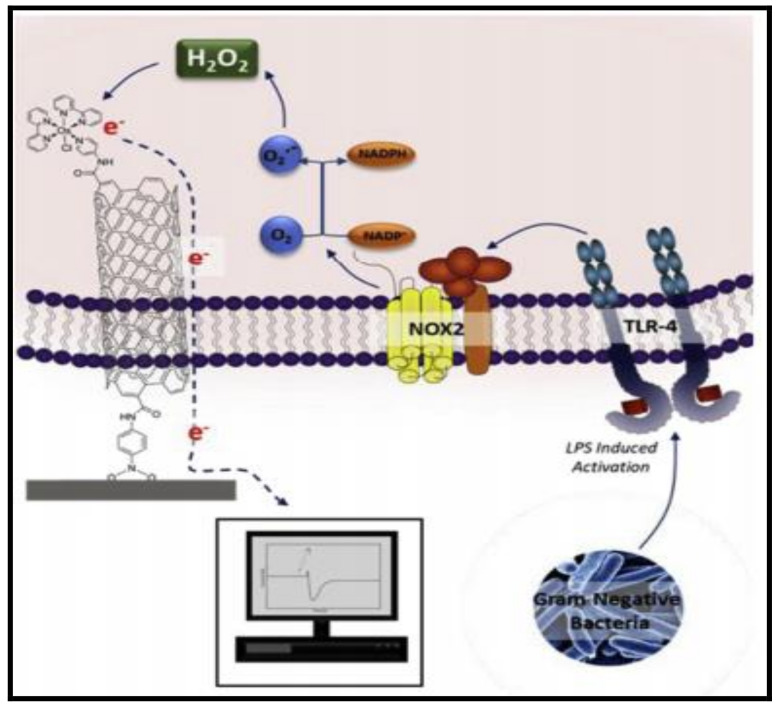
Schematic representation of pathway of detecting pathogenic bacteria through intracellular reactive oxygen species, reproduced with permission from [34] (J.M. Hicks et al., 2019).

As nanoparticle-enabled sensors provide an excellent platform for the identification of pathogenic bacteria inducing severe diseases, a large number of developments are realized in this domain. Recently, a realistic possibility of building an intracellular sensor [34] has been explored for a real time detection of reactive oxygen responsible for bacterial growth through osmium included perceiving element and the carbon nanotube as transducer that generates the signal amperometrically. The sensor produces an amperometric signal due to rapidly produced H_2_O_2_ inside macrophage cells while exposed to Lipopolysaccharides as represented by a schematic diagram in Figure 3.

The signal, generated by the varying rate of hydrogen peroxide production due to bacterial probing in the macrophage cells of mice as a function of concentration and time, was found to be different for all of the three varieties of gram-negative bacteria. These results indicate the dynamical and adaptive nature of the immune system after an infection and a similar applicability on real samples is noticed in a specific detection and morphological characterization of a toxin DNA of *Escherichia coli* through MoSe_2_ nano-urchin’s fabrication, and methylene blue as hybridization indicator, showing differential interaction [35] with repeatability and accuracy. The accuracy observed matches with the sensing platform composed of polymeric nanocapsules used in quantitatively assessing the interplay of RBCs [36] without affecting the energy of the interaction process with the aid of optical tweezers. The nanocapsules of the size 640 ± 100 nm were prepared, adsorbed on the CaCO_3_ surface, and Rhodamine labeled. When incubated with RBCs, no adverse morphological changes of the RBCs were apparent, but the native shape was retained, despite the fact that a few nanoparticles were attached to the cell membrane. This mode of bio-sensing technology is considered highly valuable, as the sensing mechanism, which is activated and mediated by nanoparticles, resembles the progression of events in the blood plasma with a low membrane binding and non-interference in the cell-to-cell interactivity. As the sensing material preserves its integrity and nontoxicity during the process, it has also proved to be one of the best methods to probe cell interactions and the toxicity effect of nanoparticles.

**Figure 4:**
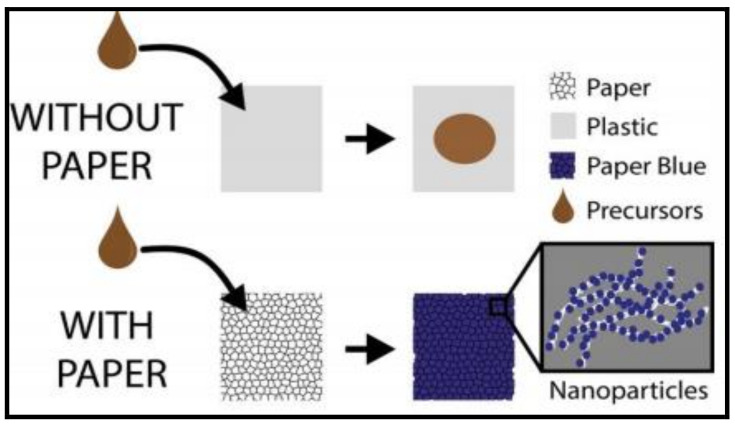
Favorable production of nanoparticles on paper as compared to other substrates, reproduced with permission from [37].

Scaling up, this innovation is also seen in the evaluation of glucose levels through commercially available, self-monitoring strips under the name Bayer Contour XT, fabricated with Prussian blue nanoparticles impregnated on ecofriendly cellulosic filter paper [37] and a non-enzymatic Ni-Pd@AC/GCE [38] electrochemical sensor that exhibits high stability and superior reproducibility even under dry conditions. The former is a green featured scaffold platform with nanoparticles of Prussian blue held on the anchor points for the reduction of H_2_O_2_ produced during the glucose oxidation. The representation in Figure 4 indicates the effectiveness of paper as a substrate with strong cellulosic fibers for the formation of nano-sized Prussian blue as compared to plastic.

The exceptional stability shown by these electrode sensors has motivated the design of P2Mo18 platforms [39] capable of bridging electron transfer across glucose dehydrogenase and a multi-wall carbon nanotube matrix in a glucose sensing setup. A low glucose saturation and a superior responsiveness of 0.198 mA mol L^−1^ cm^−2^ is achieved in a linear range of 1 m mol L^−1^ to 20 m mol L^−1^ with satisfactory 4.7 mA cm^−2^ current density with 0.2 V, mainly due to the associated multiwall carbon nanotube. The structural versatility opens up the possibility of moderating redox potential and long-term stability of the constructed sensor. A latest advancement is the design of an electrochemical glucose sensor composed of Graphene/Poly (aniline-co-diphenylamine) [40] loaded on graphene to form a stable hybrid, which exhibits high electro-catalytic activity towards glucose.

Although challenging, similar development is observed in simultaneous sensing of biological molecules, which offers an advantage of lowering the cost and time during the phase of screening. Among the variety of simultaneous estimations carried out, nanowires of RuO_2_ on CeO_2_-Au [41] fibers of nano size, operationalized through carbon nanotubes and graphite oxide composite, is a prominent sensor in quantifying serotonin, dopamine, and ascorbic acid. The setup brings out hybrid architecture of directly growing RuO_2_ nanowire on nanofibers of CeO_2_-Au providing a large surface prepared by electrospinning. The concentration of each analyte was varied separately in biological fluids along with pharmaceutical samples and analyzed with a wide detection scale and validated for tolerance and anti-interference ability. Peak potential for ascorbic acid, dopamine and serotonin remained unchanged with intensity of peak current intensity showed lower than 5.3%, 4.1%, and 3.9% decrease, respectively, compared to the first response result, which confirmed the stability of the sensor.

However, though noninvasive, these sensors are confronted with limitations, such as dependency on surface characteristics, requirement of huge volumes of statistical data, high expenses, a requirement of biological knowledge, constant monitoring of the toxic compounds that they may release, and contamination of sensing material.

These factors often result in background uncorrelated signals that contaminates the biological image quality or the microscopic biological recordings and demands certain control techniques, such as time-gating, deconvolution, multibeam scanning, signal modulation, and subtracting technique to enable high quality imaging. This is another popular subtraction algorithm-based technique [42], which is a relatively cost effective and highly effective and easily implementable method, especially for vivo imaging. The technique extends provision for realignment of light path, penetration of light into turbid biological tissues and nanoscale single molecule localization. Currently, this methodology is adapted in several areas, such as quantum sensing, expansion microscopy, phase imaging, and many others. With the advent of these promising versatile approaches, problems of doubling of acquisition time and 3D special resolution can be overcome, making biological super resolution imaging a reality.

### 3.3. Food Industry

The past decade marks the emergence of numerous sensing nanotools in food industry enabling quantification of moisture, freshness of food, extent of toxins, and contaminants. Incorporating these sensors on an industrial scale has provided a scope for minimizing food spoilage, prolonging shelf life, reduction of waste, and assessing safety from the beginning of production to the end of supply chain.

**Figure 5:**
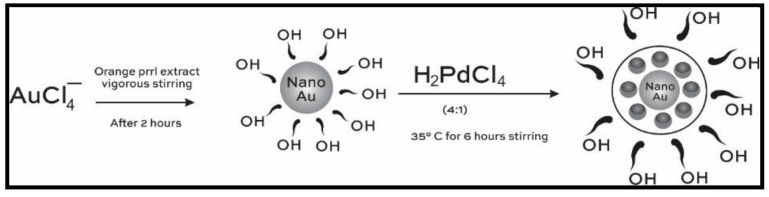
Representation of an hydroxyl group associated Au-Pd core.

Carbon nanodots multifunctional platform to determine Vitamin B2 in food samples [43], carbon quantum dots employed in identification of new coccine in food samples [44], and Au-Pd deposited gold bimetallic nanoparticles used as a signal transduction tool in colorimetrically estimating formaldehyde [45], which is a simple organic compound widely used in packaged food as a preservative and known to cause symptoms of seizures, inflammation in the throat, and sometimes may prove fatal when inhaled or ingested, are some of the classic examples. These Au-Pd nanoparticles are reported to be synthesized through a green route using an hydroxyl group of flavonoids in orange peel extract, which reduces AuCl_4_^−^ and PdCl_2_^−^ to nano-sized Au with Pd, respectively, and also to form an Au-Pd core, as represented in Figure 5. The reducing agent property of HCHO is exploited to initiate a concentration dependent color change of Au@PdNPs and cause colorimetric sensing. An excellent linearity with R^2^ = 0.991 proves the efficiency of this method.

**Figure 6:**
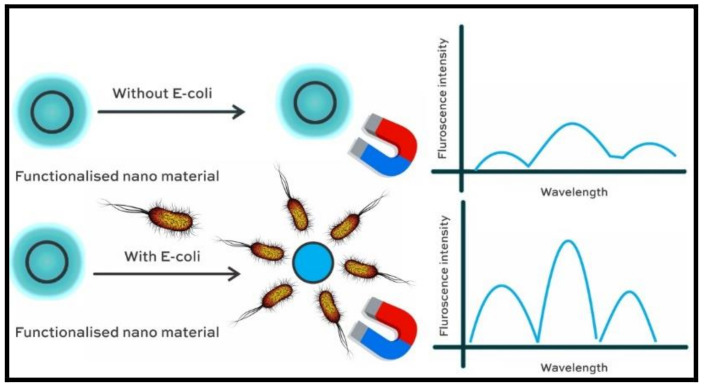
Schematic description of magnetic nanoparticle-based aptamer sensor.

One of the remarkable achievements of nano-enabled food sensors is the design of an aptamer-based sensor with a conjunction of up-conversion nanoparticles with magnetic nanoparticles [46] to quantify *E. coli* in edible matter, such as real sampling of pork meat. The method generates an electromagnetic signal due to the binding of amine functionalized magnetic nanoparticles with the target *E. coli* as represented in Figure 6**.**

The element responsible for the recognition of the microorganism is retained on the surface of an amine functionalized magnetic nanoparticle, thereby enhancing signal intensity. The study also validates the designed system in the presence of structurally similar pathogens, such as *Staphylococcus aureus* and *Salmonella typhimurium*, with the help of a fluorescence reaction at 662 nm offering a high quantum yield that ensures reproducibility and anti-interfering inertness. Further, the standard spiking methodology confirmed the applicability of the designed system in a complicated matrix through paired sample *t*-test yielding *p* > 0.05. Similar advancements in detecting and monitoring *E. coli* onsite has been devised using a Prussian blue suspended multiwall carbon nanotube embedded with a gold nanoparticle [47] system used for modifying the electrode and generate the cyclic voltamograms. Interestingly, the study adopts portable PC software that transmits data with commands via Bluetooth, performs operations on real time data through charting cyclic voltamograms, and references curves with an excellent linearity. The PC software, based on parameters such as communication, detection, online data movement, and data presentation, was implemented precisely to *E. coli* to enable the calculations on the electrochemical responses at varied concentrations. The success of the detection with high precision and reproducibility has motivated a group of researchers to develop ultrasensitive immunobiosensors to detect *E. coli* O157:H7 in milk samples [48] with the help of cadmium sulphide quantum dots entrapped in a metal-organic structure acting as signal amplifier tag. Crystalline CdS quantum dots are enclosed in ZIF-8 to create a core-shell of CdS@ZIF-8 particles that are reported to be prepared using mercaptoacetic acid as a means of capping agent. An intense amplification was achieved with a large number of such units, each labeled to the individual bacterial cell wall acting as a signal tag. The system has proved to be one of the best working immunoelectrodes with standard deviations lower than 5% and applicable to 10-fold diluted samples of milk. The approach is also suitable for sensing *E. coli* within an hour in complex natured samples of orange juice [49] through a potentiometric sensor of hydrogel nanofiber with a large surface area that transmits light and is pH sensitive. The sensor operates over the swelling and deswelling of hydrogel during the metabolic process of sugar by *E. coli* producing lactates and acetates.

Similarly, an inexpensive, simplified onsite sensing tool specific towards *Salmonella typhimurium* bacterial contamination in milk [50] is developed using unmodified AuNPs in conjunction with recombinase polymerase amplification. As the technology is highly specific, exhibits no cross-reactions with any other pathogens, and cannot be extended to the enzymes of *Escherichia coli*, it finds use in local food processing, detection, and inspection facility centers. However, the sensitivity of these devices may be reduced by the functionalization of nanoparticles used in these sensing devices. Therefore, nonfunctionalized methods, such as colorimetric detection of foodborne pathogens by nonfunctionalized silver nanoparticles aided by multiplex PCR methodology [51], are making progress in optimizing food microbial analysis. The authors report a simultaneous sensing of *L. monocytogenes*, *S. typhimurium*, and *E. coli* O157:H7, which is also apparent to the naked eye, with a detection limit of 10 pg mL^−1^, 19 g mL^−1^, and 50 pg mL^−1^, respectively.

**Figure 7:**
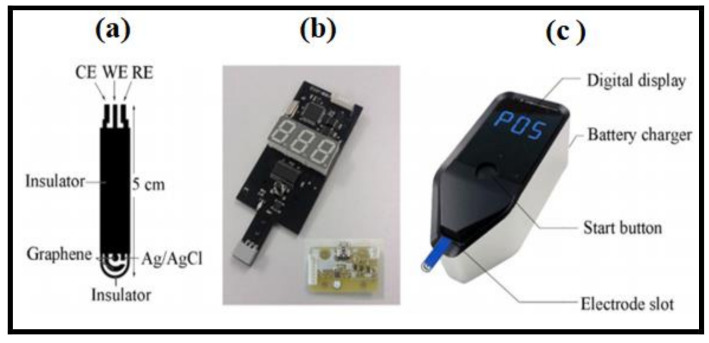
Portable mini-potentiostat (**a**) comprising a screen-printed graphene electrode, working electrode, and reference electrode; (**b**) photograph of a printed circuit board; (**c**) outer view of the portable mini-potentiostat, reproduced with permission from [53] (Jantana Kampeera et al., 2019).

Another example of engineering nano-enabled sensors with high sensitivity is the detection of electrocatalytic activity of natamycin, often used as adducts to arrest the growth of molds, fungus, and yeasts in samples of food, such as yoghurt drinks and cheese. A disposable sensor to determine the natamycin in homogenized yoghurt drinks and cheese [52] is proposed as a having wide linear dynamic range with the help of multi-wall carbon nanotubes in conjunction with the Pt doping process of CdS nanoparticles. The sensor is based on the response of electrodes through cyclic voltammetry complimented with differential pulse voltammetry techniques exhibiting a linear correlation to natamycin with the detection limit of 0.12 µM. A similar technology of screen printed electrodes subjected to isothermal amplification, which is loop-mediated, without surface modifications, is utilized in the detection platform against the Vibrio parahaemolyticus pathogen in raw sea food [53]. The system, based on a screen-printed graphene electrode, as shown in Figure 7, holds great promise for analysis of the food borne pathogen V. parahaemolyticus in the range 0.2 µM to 70.0 µM concentration, optimized at 65 °C. A portable, low power consuming, easy-to-use mini-potentiostat executing loop-mediated isothermal amplification was developed, driven by software developed by C programming.

The device generates oxidation signals of DNA bands of H33258 across the range 0.0 and 800 mV with a scan rate of 50 mV and 50 mV/s and evaluates the data through NOVA 1.10 software package.

Despite the significant role played by these sensors in the food industry towards cost savings, the techniques must fall in line with legislation requirements embracing regulatory compliance of food and hygiene norms. Furthermore, the complexity of the detecting systems must be simplified with high sensitivity and better correlation of data with the real world and computational analysis. Table 3 provides a comparative representation of various nano-based sensors in biological detection and the food industry.

### 3.4. Environmental Monitoring

Outstanding execution of performance with a high level of selectivity and reliability becomes highly essential for a timely continuous monitoring of the concentrations of environmental pollutants. Sensing processes are one of the most prime tasks in integrating the various efforts towards preventing the hazards produced by the environmental contaminants. When the sensing unit that responds to the external surrounding, or a transduction unit that translates the response to electric or optical signal, is made of a nanomaterial, it gains the advantage of tunable unique properties associated with its surface and structure.

As the focus of most current research scenarios is on the harmful particulate matter in the range of 10 microns to 2.5 microns, compact sensing platforms made of nanomaterial are becoming very popular. In this framework, electrochemical sensors with a high operational stability are in demand to detect ions [54] along with organic moieties, such as Dufulin [55] and 4-Nitrophenol, which, when released in large quantities, causes appreciable harm to the living species around. In the direction of addressing this pressing issue, a sensor constituting protonated polyaniline, boosted with graphene oxide reinforcement [56] in the form of flakes, is designed to interconnect Iron tungsten nitride and coated on to the glassy carbon electrode as a modification. Thus, the fabricated sensing nanocomposite provides a superior platform with responsiveness of 5.2 nM/18.2 nM/253.08 μAµM·cm^−2^ for identifying and quantifying the 4-Nitrophenol derivatives in the real time aquatic samples. More importantly, the modification did not suffer any degradation during the diffusion-controlled oxidation/reduction shuttling between 4-(hydroxyamino) phenol and 4-nitrosophenol, but perfectly indicated the electron communication through the redox probe in cyclic voltammetric analysis. Numerous equivalently popular examples of synergic effects of nanocomposites with environmental relevance are emerging in large numbers. For instance, a novel nanocomposite MnFe_2_O_4_/ZrO_2_ [57] has shown decolorization response towards methylene blue organic dye through adsorption and desorption at an optimum concentration of 20 ppm. The technique, which is validated at different concentrations using industrial waste water, has proved to be an efficient sensing methodology and may find potential to be extended in monitoring organic dye toxicity in surface water resources.

**Figure 8:**
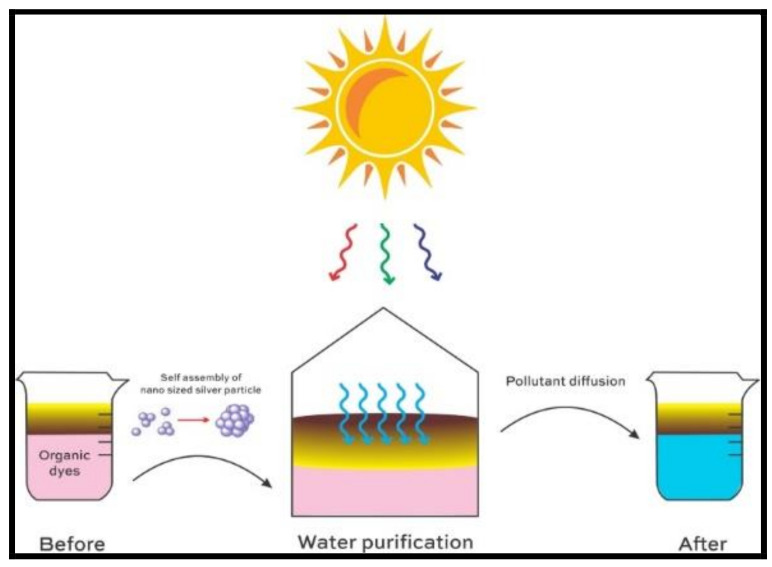
Sensing the toxic organic dyes by asymmetric nano-sized silver particle based plasmonic structures.

A similar application of nanocomposite is Graphene Oxide–Fe_3_O_4_ in the analysis of phenazopyridine [58] in aquatic systems, wherein a high performance of adsorption and high energy of interaction across phenazopyridine and Graphene Oxide–Fe_3_O_4_ residue is observed. The designed system has an advantage of high surface area, high recovery percentage, and, hence, also finds a cost-effective industrial application validated by density functional theory. Generally, these composites require an optimization of adsorbent dose, pH, contact time, temperature, and a well-defined concentration range in ppm and seldom are overlooked when compared to dual functioning materials, such as asymmetric nano-sized silver particle based plasmonic structures [59] utilized in sensing the toxic organic dyes in water and, simultaneously, providing an off-grid solution for quality examination and solar water purifying units. The sensing system is verified with Direct Red 23, Direct Blue 15, and Direct Black 19 and has proved to be highly effective, offering a pronounced spatial uniformity, long term stability, and durability. Surface enhancement Raman spectroscopic measurements further validate the responsiveness of the system towards solar vapor generation for different solar irradiation intensities arising from close-packed Ag nanoparticles, as shown in Figure 8.

#### Plasmonic Structures

These integrated dual functioning systems form an impressive basis for further progression of voltammetric pollution detection devices undergoing a modification using Fe_3_O_4_-NPs/HMPF_6_/CPE [60]. The authors in this elaborative article clearly examine the chronoamperometric patterns to simultaneously determine two water contaminants 2-phenylphenol and 4-chlorophenol with an application of voltage of 600 mV over a range of 100 μM to 300 μM. The oxidation response peaks of both of the contaminants is well apart without causing interference to each other and lead to an equation of linear regression indicating a diffusion-restrained process. The remarkable stability and an excellent sensitivity of 0.0201 μA/μM and 0.0093 μA/μM, which agrees with the results of the calibration curve, reconfirm the sensor’s capability of specificity.

Despite these successful techniques, a great demand is witnessed for the design of sustainable materials, which is met by graphene-based materials. Some forms of graphene, such as Graphene Aerogels reported by Yusik Myung et al. [61], have indirectly contributed to the depletion of environmental pollution through storage devices with high efficiency. These are well-designed lightweight devices with 3D intertwined frameworks derived from biomass with versatile characteristics that enhance the kinetic rate of ion diffusion and, thereby, the specific energy in symmetric supercapacitors with an extremely high energy density of 56.80 Wh kg^−1^ and a high-power density of 620.26 kW kg^−1^. A remarkable feature of this material is the green synthetic route adopted, mediated by raw pear for hydrothermal synthesis with subsequent carbonization. On further activation, an increase in specific area from 1001 to 2323 m^2^ g^−1^ was noticed, which made it an excellent adsorbent towards toxic organic cationic/anionic dyes. XPS spectral analysis, along with Raman analysis, explores the graphitic structure possessing unique lattice fringes with the extent of activation, while Nitrogen adsorption/desorption tests indicated the porous nature and a large surface area available for the intermolecular adsorption. However, as aerogels are prone to heat energy loss, a thermodynamically more feasible emerging technology of hydrogels that can respond to stimuli would be more appropriate for a regulated delivery of agrochemicals. Generally, agrochemicals are aromatic in character and are sparingly soluble in water, which makes the entrapment or attachment of molecule cumbersome.

Xiaobang Hou et al. have successfully formulated cellulose nanogels that possess reversible sol gel transitions [62], which can overcome the difficulties of loading capacity. This multiresponsive, stimuli-responsive, and redox responsive agro carrier, is built on the model containing sodium carboxymethyl cellulose, with hydrophobic branches, facilitates the loading of lipid-soluble salicylic acid, which is a well-known phytohormone capable of regulating the conception of ions and photosynthesis in plants. The capacity of salicylic acid to influence the pathogenesis associated gene-1 is exploited to mimic a slow release of agrochemicals with a simultaneous soil remediation.

Furthermore, fluorescent carbon dots have for a long time shown good potential for sensing in environmental remediation, which is proved by a recently fabricated biocompatible carbon dot sensor [63] used in wastewater treatment. The prepared carbon dot sensing material has an advantage of transforming the highly infectious environmental contaminant agarose waste during the course of fabrication. The structure of the generated carbon dots was validated by HR-TEM image, XRD pattern, BET isotherm, EDX spectra, DSC heating profile, and FTIR spectrum. HR-TEM showed the microstructure of 2–10 nm sized dispersed carbon dots with weak lattice spacing and XRD across 14 to 57° with graphitic nature confirmed by peak centered at 45.2° corresponding to characteristic planes in carbon dots. The desorption points in the spectra indicates a non-porous nature, shows high composition across 2–4 keV in EDX, DSC curve displays the effect of heating with glass transition and melting temperature to be 115.1 and 132.9 °C, respectively, and, finally, the surface functional group confirms the surface functional groups.

The so-prepared carbon dots were found to be highly biocompatible when tested for antibacterial activity on gram negative *E. coli*, and gram-positive *S. aureus*, antifungal activity on *Laurilia taxodi* strains, anti-proliferative or cytotoxicity activity on *Vigna radiate*, and genotoxicity using Allium cepa. The carbon dot substrate acts as a sensory device towards cations and anions through fluorescence emission and was implemented in the analysis of tap and lake water containing Zn^2+^ and CO_3_^2−^ ions.

A similar colorimetric detection is extended to fluorimetric mode by M. Anju et al. which utilizes grapheme oxide–fluorescein blend [64] for selective sensing of Fluoride ions. This technology is helpful in regulating the concentration of fluoride ions, which in exceeding levels of concentration in water can cause serious symptoms of dental caries, kidney disorders, and may prove even fatal in many cases. The sensing platform works on the principle of replacing fluorescein by fluoride ions, in grapheme oxide–fluorescein blend, which leads to enhancement of optical response. The usage of both colorimetric and fluorometric methodologies enables quick and stable response in diverse complicated environments of real water samples at low concentration of 3 ppm. The sensing assembly consists of fluorescein anionic dye clinging on to the reduced graphene oxide sheets via non-covalent π-π interactions, hydrogen bonding forces, and dispersive forces with a moderate repulsive force. The highly charged F^−^ ion facilitates the interaction and subsequent replacement of fluorescein, thereby causing highly selective sensing.

**Figure 9:**
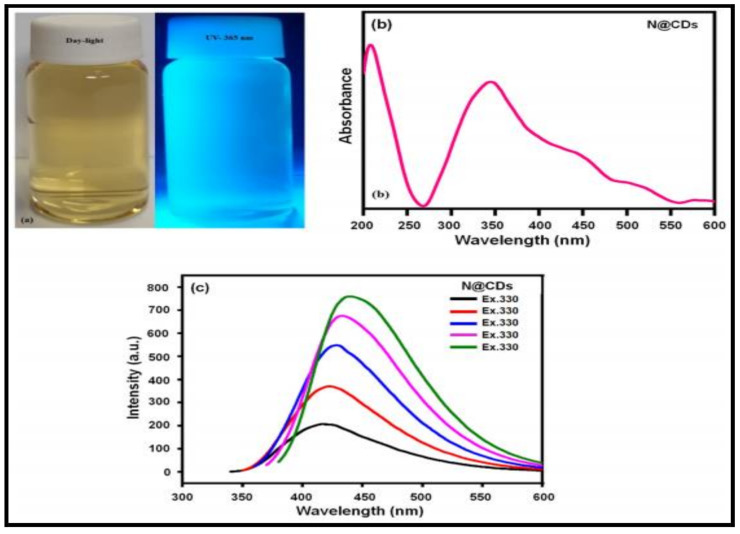
Nitrogen doped carbon dots (**a**) exhibiting fluorescence; (**b**) UV–visible absorbance; and (**c**) fluorescence at various emission wavelengths, reproduced with permission from [65] (Moorthy et al., 2021).

Another interesting report on the use of fluorescence quenching (Figure 9) is through carbon dots doped with nitrogen that detect Eu3 + ions [65] by entrapping them across the amine and phenolic groups on its surface. These nitrogen doped carbon dots in solution, when excited to 365 nm, show an intense blue fluorescence (Figure 9a) as compared to day light, a strong UV–visible absorbance (Figure 9b) at 205 nm corresponding to *n* − σ∗ in lone pair electrons of O and N, a broad peak close to 350 nm owing to *n* − π∗, and also excitation dependent fluorescence (Figure 9c) across 350 to 550 nm. Furthermore, the electrostatic interaction is reflected by the decrease in fluorescence intensity and considerable change in IR spectra.

Oluwatobi et al. have also made a successful attempt in colorimetrically sensing the mercury ions incorporating silver nanoparticles prepared with the aid of hyacinth plant leaves [66]. The sensing system is designed with a simple working procedure where the solution of silver nano particles is introduced to a solution of mercury and the peak intensity is monitored after two minutes of stirring using UV–Vis spectroscopy with concentration as small as ranging from 10^−4^ to 10^−8^ M of Hg^2+^ solution.

**Figure 10:**
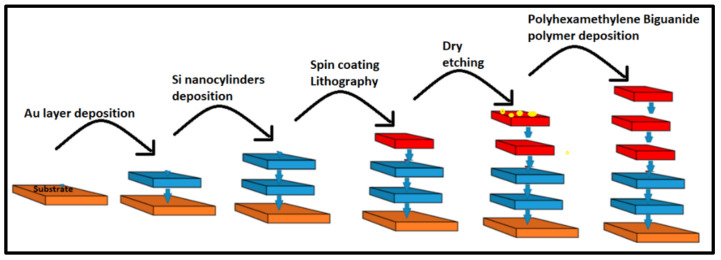
Sequence of fabrication steps involved in CO_2_ gas sensors.

Some of the most vital devices that ensure a clean environment and are gaining much interest worldwide are the nano-equipped gas sensors for the detection of NOx, NH_3_, O_3_, CO, and CH_4_, etc. These nano-enabled gas sensors, owing to their sensitivity, highly ordered structures, large surface area, uniform distribution, and deposition by spray coating or spin-coating techniques [67], do not demand chemical reaction cycles. With regard to monitoring of the concentrations of environmental pollutants, oxide-based sensors are well known for their improved sensing performance as they offer an excellent surface to volume ratio, easy doping, are stable to a wide range of operating temperatures, and extend a highly regulated dimension. A highly selective ZnO-Nanoribbon device [68] with two probes and zigzag edges is designed that shows enormous sensitivity to predominant H_2_, O_2_, and CO_2_ molecules. It shows highly promising adsorption and transport properties in accordance with density functional theory and Green’s function (NEGF) formalism. It is apparent from the data that H_2_ dissociates leading to enhanced conductivity, while O_2_ and CO_2_ have weak π-bonds that break triggering a response on the substrate. Another response efficient material is fabricated and realized to have high sensitivity with metamaterial based functional host polyhexamethylene biguanide polymer [69] loaded upon silicon nano-cylinders periodically arranged on a slender wafer of gold foil, which is highly responsive to polarized light oriented across the X-Y axis, as depicted by Figure 10**.**

The variation of refractive index of the host as a function of CO_2_ concentration is registered adapting wavelength interrogation method with a sensitivity of 17.3 pm/ppm for a gas concentration of 434 ppm. However, the area of gas sensing is too vast to be elaborated here, but it is worth mentioning that the majority of the gas sensors are governed by precursor, pressure, and temperature and, hence, demands a firm design and numerical investigation. These milestones of nano-based environmental detectors, which are consistently used in detecting and analyzing organic and inorganic toxins through surface composition, size, dissolution, and aggregating capacity, are paving the way for an environmental cleanup.

### 3.5. Nano-Based Sensors in Pharmaceuticals

One of the main medical applications of nanosensors is in the field of pharmaceuticals, where they are deployed in the form of optical, electrochemical, and chemical nanosensors. Emerging applications of nanomaterial, owing to their sensory performance in the analysis of pharmaceuticals, has many advantages in various phases starting with drug designing, formulating, stability testing, to toxicology assessment, clinical trials, and also biomimicking. It is a relatively recent realization that nanosensors, owing to their large surface area, minimum toxicity, and favorable kinetics, could bring a new generation of probing technology in all of the different aspects of present pharmaceutical analysis. The outstanding physicochemical properties of nanostructures facilitate the easy fabrication and modification of surface functionality [70], enabling a unique interface for the recognition and generation of electronic signals with an excellent limit of quantification [71]. One of the remarkable achievements of nano-enabled sensors applied to pharmaceuticals is the drug-association investigations where simultaneous analysis leads to identification of substances interfering with the stability of the drug and cross-interactions amid active principles of the formulation.

In a recent study, a dual nano-donor coupled to single nano-receptor aptasensor connected to bi-color FRET [72] was successfully used to simultaneously detect and quantify two highly harmful mycotoxins aflatoxin B1 and ochratoxin A with a single run through target-induced fluorescence restoration.

**Figure 11:**
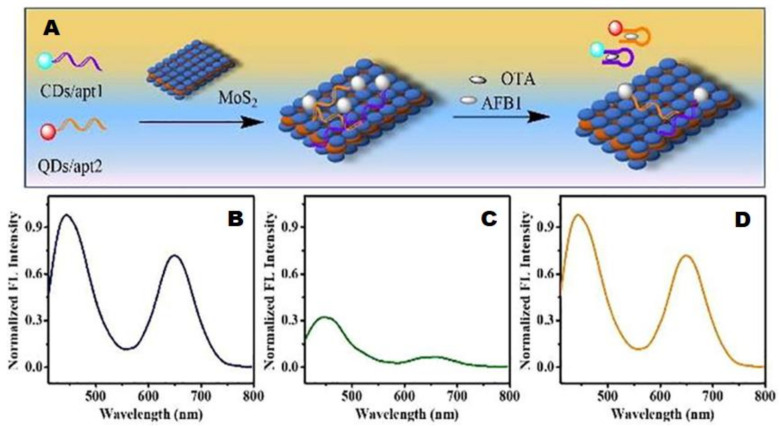
(**A**) Schematic representation of fabrication and sensing of aptasensor, fluorescence spectra of blend CDs/apt1 and QDs/apt2; (**B**) before bioconjugation; (**C**) after bioconjugation; (**D**) incubation with 20 mg/mL of toxins aflatoxin-B1 and ochratoxin A, reproduced with permission from [72] (Jing Qian et al., 2020).

Figure 11A shows MoS_2_ nanosheets with two distinctive donors carbon dots and CdZnTe quantum dots. These nanosheets are capable of acting as single nanoacceptors paired to multiple nano-donors.

Aflatoxin-B1 and ochratoxin A aptamers are coupled to carbon dots and CdZnTe quantum dots, whose emissions are displayed in Figure 11B, at 447 nm and 650 nm. A considerable decrease in intensity of emission is observed in Figure 11C due to the introduction of MoS_2_ nanosheets, which indicates an absorption on nano-acceptor surface leading to a bioconjugate species [carbon dots/aflatoxin-B1-MoS_2_-quantum dots/ochratoxin A] through stacking interaction. Further, the tags are released from MOS_2_ nanosheets, leading to the recovery of fluorescence shown in Figure 11D. In summary, the sensor is based on a simple procedure of incubating both the toxins in varying concentrations and gradually increasing the normalized fluorescence strength of the designed aptasensor system, which gives the limit of detection through two linear relationships in the concentration range 0.1, 1 and 5 mg mL^−1^ and 0.1, 1 and 3 mg mL^−1^ for aflatoxin-B1 and ochratoxin A, respectively.

**Figure 12:**
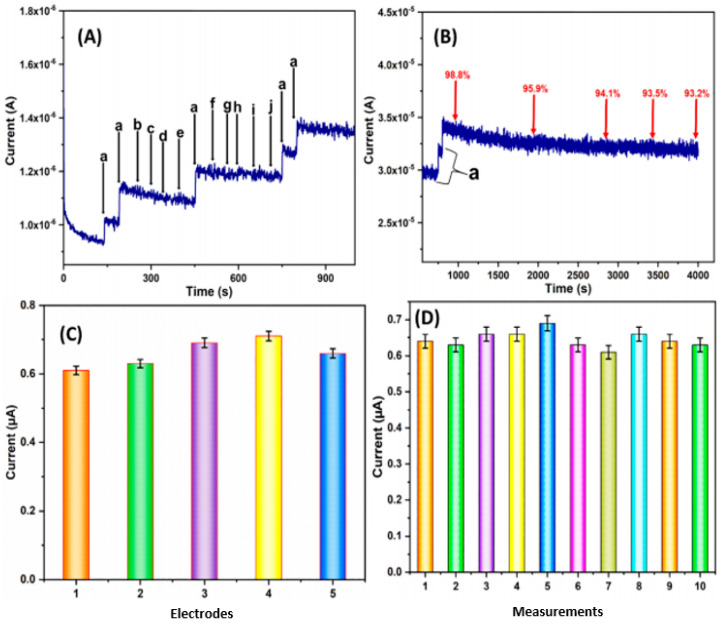
(**A**) Amperometric response at −0.42 V on adding various adducts with (**A**) nitrofurantoin; (**B**) amperometric response of modified electrode to 30 µM nitrofurantoin; (**C**) repeatability; (**D**) 10 successive amperometric measurements for 30 µM nitrofurantoin, reproduced with permission from [74] (Sethupathi et al., 2019).

Simultaneous biosensing has also been attempted for the first time by Shokoufeh Soleimani et al. [73] on doxorubicin, daunorubicin, and idarubicin drugs, which were impossible to detect through conventional cyclic voltametric methods that led to overlapping signals under simulated physiological conditions. Hence, electrochemical impedance spectroscopy data were generated on a multi-walled carbon nanotube and bovine serum albumin modified electrodes, which were used to build multivariate calibration first-order curves through partial least squares analysis in combination with impedimetric data. The strategy was validated on a group of fifteen serum samples of known concentration and ten samples of random serum concentrations with complex matrix, which requires graphical plotting, statistical methods, simulation using first order algorithms, predicting root mean square errors, and relative error. In another remarkable socially significant study, a nitrofurantoin sensing [74] technique is developed through chitosan hydrogels embedded on multiwalled carbon nanotubes prepared by ultrasonic method. The multiwall carbon nanotubes were functionalized by nano-sized hydroxyapatite by sonication at 37 kHz and, the so developed hybrid, with a large surface region, serves as a sensitive tool for modification of the electrode towards detecting nitrofurantoin within the range of 0.005–982.1 µM. The sensing specificity is such as Furazolidone, Uric acid, 3-nitroaniline, dopamine, and many other metal ions. A predominant response of nitrofurantoin was notable from Figure 12A, while the response of other added interfering compounds was negligible. The corresponding amperogram in Figure 12B shows retentively of 98.8, 95.9, and 94.1% of initial steady-state current response at an interval of after 900, 2000, and 2900 s indicating a good operational stability and its affinity towards the electrode. An excellent current response towards nitrofurantoin with standard deviation of 4.12 in Figure 12C and detection of 3 µM nitrofurantoin, with the aid of 10 successive amperometric measurements in Figure 12D, reaffirms the specificity of the designed electrochemical system.

An excellent performance of this sensor with a detection limit of 1.3 nM has contributed enormously in monitoring such highly reactive antibiotics that destroy good bacteria, promoting resistance to pathogens and debilitating the immune system. Since the ban of nitrofuran in 1984, many such promising sensors are fabricated, among which exceptionally remarkable nitrofurantoin and nitrofurazone thin film sensors [75] constituting {[Eu2(BCA)3(H_2_O)(DMF)3]0.5DMF·H_2_O}nEu-2,2′-biquinoline-4,4′-dicarboxylate) are noted ones in the form of compact thin film. The sensor is tested in Pearl River samples along with bovine serum samples and has been shown to be highly sensitive to antibiotic-induced toxicity in livestock. The compound is recognized by Co_3_O_4_ nanowire tree-like arrays anchored on stainless steel mesh forming a 3D metal-organic ligand framework of adaptable pore size. The sensing ability of the compound arises due to its ability to transfer the electrons from the lowest occupied molecular orbital of high energy to the least occupied molecular orbitals lacking molecular orbitals of antibiotic molecules, which was validated by subjecting the thin film to the concentration-dependent luminescence quenching effect where distinctive emissions of Eu^3+^ are exhibited due to electric dipole transitions from 5D0 to 7FJ with J = 1, 2, 3, 4 leading to a prominent peak at 615 nm.

A comparable sensing sensitivity is achieved by nano MnFe_2_O_4_ [76] modified graphite electrode in a neutral medium, which magnificently exhibits an electrochemical response towards paracetamol and D-glucose at concentrations as low as 1 mM to 5 mM. The electrical interaction is characterized by the cyclic voltammetric curves of the sensing material, which intensifies and changes its position on exposure to the analyte. Paracetamol is reported to result in oxidation peaks at −0.11 and 1.0 V jointly, with two small reduction peaks at 0.63 and 0.02 V, and glucose yielding a broad anodic peak at 0.63 V, along with cathodic reduction peak at 0.21 V. The authors have also extended the activity of the oxide to Ce-doped MnFe_2_O_4_ [77] with excellent activity and one of its kind to exert the sensing activity in NaNO_3_ electrolyte aided neutral media. The technique, due to its uniqueness, has attracted the possibility of being metamorphosed in nanoelectronic sensing devices.

In conjunction with these developments, nano-enabled in vitro release detection is emerging in medical procedures and would be more appealing with the incorporation of natural product-based moieties. Recently, copolymerized methacrylate derived sugarcane bagasse cellulose based multiresponsive nanogel [78], which is reported to be used for controlled release of doxorubicin hydrochloride, is highly responsive to temperature and pH. The pH sensitivity is associated with electrostatic interaction across methacrylated monocarboxylic sugarcane bagasse cellulose and doxorubicin hydrochloride, which is weakened at low pH, thereby supporting the drug release. The release effectiveness of drug-loaded nanogels was found to elevate with decreasing environmental pH, and around a pH of 4.0 the release effectiveness reached 50%. These drug release systems may be coupled with a pH detector developed for real time monitoring, based on green synthesized carbon dots [79] whose emission intensity is pH dependent. The changes in fluorescence color, caused due to variation of pH, is noted and evaluated by the image analyzer.

While these new developments add impetus to pharmaceutical analysis and assays, many challenges, such as incorporating algorithms compatible with optical and electronic sensor architecture towards increasing the sensitivity, are confronted in making them more accurate and affordable.

### 3.6. Nano-Based Sensors in Diagnostics

As the size of most biological molecules and cell organelles fall within the nanometer scale, the extension of nanotechnology to diagnostics has brought revolutionary changes in the form of biomolecular detectors [80] DNA nanomachines [81], imprinted polymers [82], DNA tweezers [83], and enabling biological tests [84] to be more sensitive and quicker, which is most demanded in clinical laboratories. Nanomaterials, due to their smaller size, can drastically enhance one-to-one interaction across the analyte and signal-generating target biomolecules. Many limitations in conventional diagnostics, such as time-consumption, laborious processes, and requirement of expert in diagnosis for analysis, is overcome by current upgraded diagnostics with the intervention of nanosensors playing the role of multifunctional platform. As a result, molecular technologies, such as genomics, transcriptomics, and metabolomics, have developed rapidly over the decades with sensitivity reaching the level of a single molecule.

**Figure 13:**
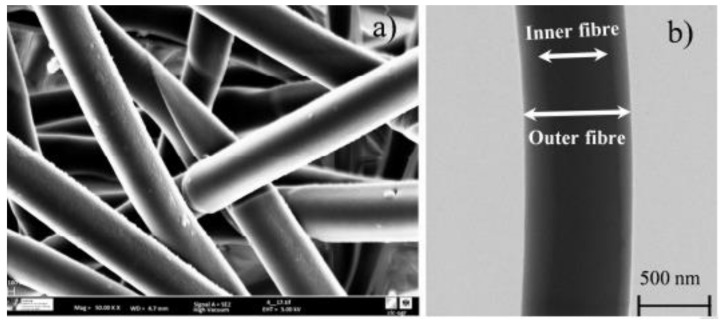
Pictures of (**a**) SEM; and (**b**) TEM of the multifunctionalised coaxial membrane, fabricated using PMMA as inner fiber and PdTFPP PolymBlend as outer fiber, reproduced with permission from [85].

A remarkable milestone in the development of such molecular technologies is by Teresa Ramon et al. [85], who have developed a multifunctional sensing material towards oxygen sensitive dyes through the inner fiber and an outer fiber carrying functionalization responding to oxidase enzyme. The coaxial fibers of 680 and 530 nm were fabricated by co-electro spinning using two different solutions flowing at different rates, optimum injector and collector voltages. The regularity and homogeneity of the resulting fibers are apparent from SEM and TEM pictures in Figure 13.

This material is capable of optical oxygen transduction, which is tested on determining the concentrations of uric acid contained in a serum sample in conjunction with a covalently immobilized model enzyme “Uricase” by varying the reaction parameters. As the sensing material is highly hydrophobic, the authors have utilized a polymethyl methacrylate hydrophobic environment to generate emission and excitation spectra with different oxygen dyes as the coaxial material. The results are compared by incorporating diverse dyes with respect to a classical membrane.

A similar immobilization of enzymes microbial lipase, well known alcohol dehydrogenase, and highly reactive glucose oxidase is applied onto a modified glassy carbon electrode surface by D. Zappi et al. [86] in building a portable, disposable amperometric biosensor finding applications in the agribusiness sector, clinical analysis, and environmental protection. The modification was achieved with an ecofriendly generation of IV ionic liquids at room temperature constituting cationic choline and anionic amino acids on carbon nanotubes, nanogold, and a purified form of graphene to provide a high contact area. An intense amplification of the amperometric signal with good reliability was evident when applied on real life samples of extra-virgin olive oils, beverages containing alcohol, and glucose containing food matrices. The development of such sensors has the potential to be used as user friendly multi-analyte quantitation and, hence, has inspired diagnostic strategies in sensing cardiac troponin using whiskered nanofiber implanted with carbon nanotube. The combination successfully served as an electrochemical immunosensor tool to detect an important protein troponin [87] in concentrations ranging from a normal person to patients with myocardial infarction with acceptable standard deviation. The network of carbon nanotubes provides a percolating pathway for electronic movement and was confirmed using tunneling electron microscopy micrograph. Electrochemical investigations exhibited increased peak current consistently over varying scanning cycles due to the electro catalytic activity of several segments on the exterior of conductive nanofibers. The data provided by these researchers indicate that the new sensor is a favorable candidate for rapid diagnostics.

Another proposed immunosensor to detect mycotoxin zearalenone [88] uses a fabrication of layer-by-layer congregation of composite material bound to nanogold at carbon screen printed-electrodes. In addition, the electrodes are enriched with a uniformly dispersed multi-walled carbon nanotube or polyethyleneimine. All these electrochemical immunosensors provide a good alternative for conventional materials and will soon find a place in the commercial market.

In parallel to these reports, many papers are published on sensors in diagnostics using nano-hydroxyapatite, which is a well-known phosphate mineral biomaterial with far reaching material properties in dentistry. The demineralizing effects due to free Ca finds a major role in treating caries and dental erosion. Recently, calcium hydroxyapatite, whose conducting properties are due to the moving protons across neighboring hydroxyl ions, is doped with graphite to trace alcoholic vapors [89]. The structure of sensing material is confirmed by a series of characterizations using XRD, SEM, and a suitable sensing mechanism, was interpreted where O^2−^ species on the surface of hydroxyapatite encounters alcoholic vapors forming CO_2_ and H_2_O along with liberation of electrons, which can produce an electric signal. The signal originates due to conduction of two neighboring OH^−^ produced by

OH^−^ + OH^−^ ↔ O^2−^ + H_2_O

The so produced O^2−^ reacts with alcoholic vapors liberating electrons, which results in the signal.

CH_3_OH + 3O^2−^ ↔ CO_2_ + 2H_2_O + e^−^

When combined with graphite, the surface to volume ratio increases, which bestows a large number of active reactive sites. However, the drawback of this sensing substrate is its dependency on adsorption/desorption equilibrium, which is overcome by the recently developed sensing platform constituting TiO_2_^−^ Hydroxyapatite nano-composite [90]. The authors report a mechanism where oxygen species are replaced by alcohol molecules and O^−^ ions decreases in number. Thus, electrons are released back to TiO_2_, thereby enhancing its conductivity. Despite all of these remarkable developments, the challenges associated with nanosensors in diagnostics still persists with many limitations in system integration, automation, signal amplification, and standalone platform stability.

## 4. Sensing Mechanism

The working of a sensor is largely dependent on fundamental transduction mechanisms to produce the signal and also is dependent on external connections, mode of energy transfers [91], and signal processing [92]. Generally, sensors are driven by electronic detection on electrodes or modified electrodes, physisorption-chemisorption, selective absorption, absorbance, fluorescence, surface modification, redox reaction, and charge transfer across surface anchored material–target. A few mechanistic aspects are explained briefly with illustrations under the following subheadings.

### 4.1. Physisorption-Chemisorption

**Figure 14:**
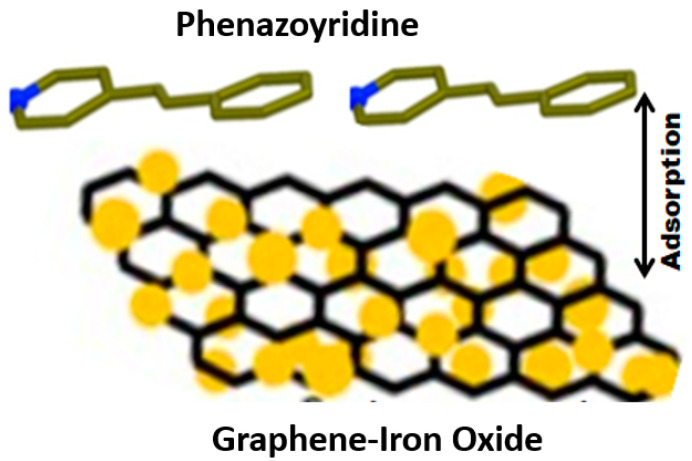
Adsorption of phenazopyridine residues on graphene–iron oxide.

Physisorption generally is an exothermic reaction occurring at lower temperatures, while chemisorption is an endothermic reaction occurring at higher temperatures and, hence, temperature regulation governs the efficiency of these sensors. For example, a thick depletion layer ruling the carrier transportation along with electron concentrations and, thereby, the resistance, is generated when O_2_, O_3_, or oxides of nitrogen is adsorbed on n-type sensing material [93]. A similar real life example is the adsorption of harmful phenazopyridine residues on graphene–iron oxide [58], which provides a large surface area for adsorption and is highly regenerative. The interaction is well explained by density functional theory, deciphering a parallel adsorption of phenazopyridine molecule on the terminal Oxygen and Iron of graphene–iron oxide, as indicated in Figure 14.

Recovery, non-deformation of substrate crystal structure, repeatability, and high sensitivity are the major advantages of sensors based on adsorption process and, hence, considered to be a valuable sensing technology.

### 4.2. Surface Modification

Surface modification is a multistage process that has drawn the attention of the researchers as it can extend the process of sensing to a molecular level and create new sensing modalities. It often includes formation of a probe layer through functionalization, interfacial layer, or passivizing the sensor surface.

In this regard, an in situ soil moisture sensor is a good example where high capacitance is achieved by orientation and interfacial polarization caused by the influence of charges on MoS_2_ nanomaterial surface [24] on the water molecules absorbed. The surface recovers to low capacitance with the lowering of water content where a negligible influence of temperature and ionic concentration is observed. In addition, adjacent water molecules exchange protons, transforms water molecules to hydronium ions, results in ion transfer and, hence, an abrupt elevation in capacitance monitored by impedance analysis.

**Figure 15:**
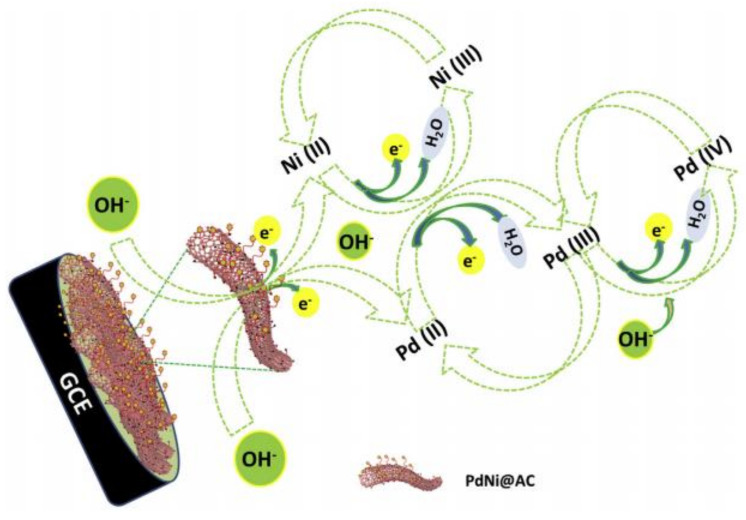
Mechanism involved during activation of Ni-Pd@AC/GCE surface in NaOH solution, reproduced with permission from [38].

Furthermore, chemical surface modification of electrodes has driven the development of sensors owing to their excellent detection limit and a wide linear range of sensitivity. A glassy carbon electrode coated with activated carbon incorporated monodisperse nickel-palladium alloy nanocomposite [38] used in the detection of glucose is a classic example. Glucose is quantified by the electrodes in the form of anodic and cathodic peak current of cyclic voltammogram. The surface modification achieved here using a nanocomposite was successful in providing a synergetic effect and a larger surface area that permits a greater charge flow reflected in the increments in the peak current. As shown in Figure 15, Ni (II) and Pd (II) existing during anodic scan is transformed to Ni (III), Pd (III), and Pd(IV) from Pd (III) at higher potentials. During cathodic scan, Ni (III) and Pd (III and IV) are reduced to Ni (II) and Pd (II), thereby generating the voltammogram.

### 4.3. Colorimetric Detection

Colorimetric sensing approaches have been highly successful owing to their simplicity, selectivity, and cost-effectiveness. The strategy relies on the changing color of a sensing material on interacting with the analyte through absorption, adsorption, complexation, or van der Waal’s forces. As often these interactions occur on the surface of the material, nanomaterials with large surface areas have promising roles to play in the area of sensing technology.

In particular, colorimetric sensing using metal ions has gained high popularity due to its instantaneous response, but often requires modification on the surface of the metal particle. AuNPs has attracted the attention of researchers due to its potential of responding to the changes in the refractive index of the medium and facilitating the interparticle surface plasmon coupling.

Generally, the preparation of AuNPs is achieved by the steps represented in Figure 2, where a core of nanoparticles is gradually surrounded by a shell of inert metal.

As an illustration, Au@PdNPs prepared by a green route involves the flavonoids in orange peel extract reducing the ${\mathrm{AuCl}}_{4}^{-}$ and ${\mathrm{PdCl}}_{2}^{-}$ to a core of Au nanoparticles and Pd shell, respectively, via the following reactions [39].

(1) $${\mathrm{AuCl}}_{4}^{-}+3\mathrm{HO}-R\to \mathrm{Au}+3O=R+3H^{+}+4{\mathrm{Cl}}^{-}$$

(2) $${\mathrm{PdCl}}_{4}^{2-}+2\mathrm{OH}-R\;\mathrm{Au}\to \mathrm{Au}@\mathrm{Pd}+2O=R+2H^{+}+4{\mathrm{Cl}}^{-}$$

**Figure 16:**
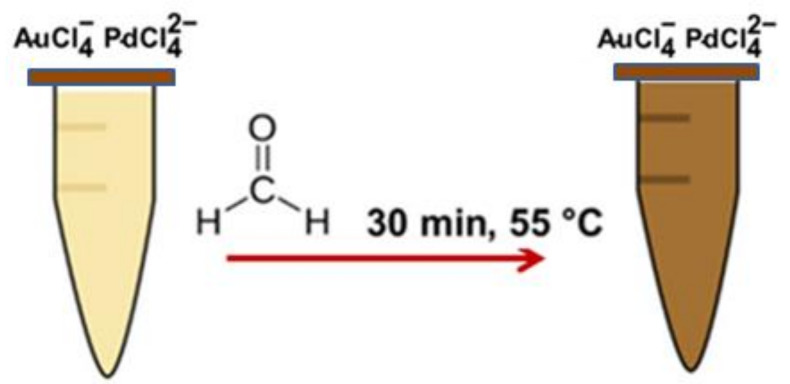
Colorimetric sensing with Au@PdNPs as a probe.

The sensing properties of the prepared Au@PdNPs were validated by comparing its response to HCHO taken as a reducing agent with simple Au nanoparticles. It was observed that Au@PdNPs showed a higher absorbance and an intense color change from light to dark brown (Figure 16) than Au nanoparticles as per the following reaction [45].

$$5\mathrm{HCHO}+{\mathrm{AuCl}}_{4}^{-}(\mathrm{excess})+{\mathrm{PdCl}}_{4}^{2-}(\mathrm{excess})\to 5\mathrm{oxidized}\;\mathrm{HCHO}+\mathrm{Au}@\mathrm{Pd}+5H^{+}+8{\mathrm{Cl}}^{-}$$

### 4.4. Electrochemical Reduction

**Figure 17:**
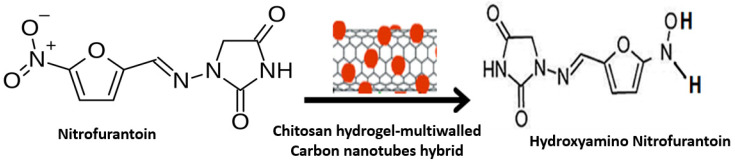
Chitosan hydrogel-multiwalled carbon nanotubes hybrid assisted. Electrochemical reduction of nitrofurantoin to hydroxyamino nitrofurantoin.

The process of electrochemical reduction is a popular selection to base a sensing strategy, especially in conjunction with nanoparticles. The process generally involves a dissociative electron transfer that may assist electro catalytic, photo electrochemical, or in the formation of activated radicals. The example that follows is a unique synergistic electro catalytic activity of the hybrid chitosan hydrogels on multiwalled carbon nanotubes on glassy carbon [74] exerting its action in detecting nitrofurantoin to an accuracy of 0.16 µA·µM^−1^·cm^−2^. The composite electrode is involved in the transfer of four protons and electrons, as shown in Figure 17, converting nitrofurantoin to hydroxyamino nitrofurantoin with the aid of a unique structural feature of carbon nanotubes offering a positive potential upon carbon.

### 4.5. Fluorescence Quenching

**Figure 18:**
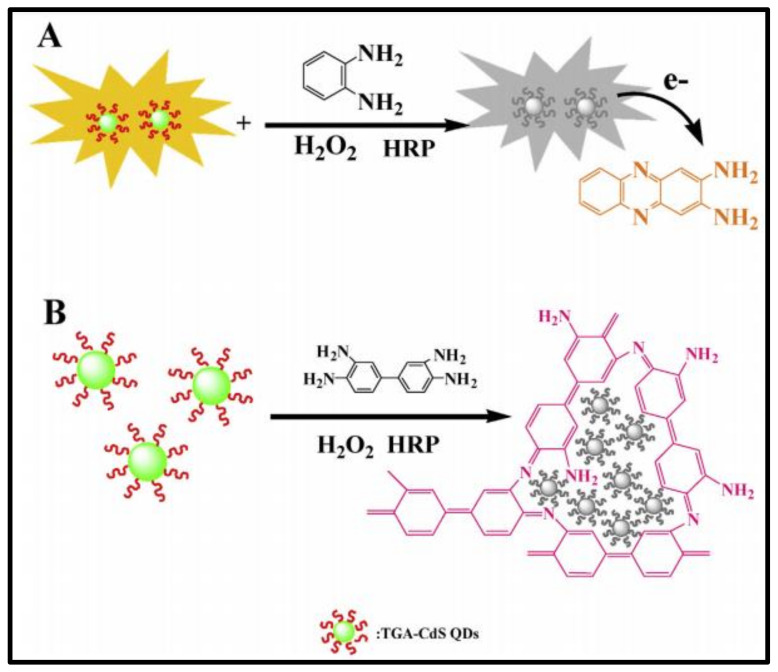
Interaction between thioglycollic acid-capped quantum dots and the oxidized o-phenylenediamine (**A**); or the oxidized diaminobenzidine (**B**), reproduced with permission from [94].

Fluorescent probes are an attractive sensing tool in the area of biotechnology owing to their luminescent properties, specificity, and photo stability. Coupling with biomolecules increases its potential uses in biological detection. A wide range of molecular interactions, such as rearrangements, complexation, and energy transfer, can cause fluorescence quenching that serves as an effective molecular probing strategy. A typical example is a glucose sensing system based on the fluorescence quenching of thioglycollic acid capped quantum dots –bienzyme hybrid system [94]. Lifetime measurements could distinguish between dynamic and static quenching that made elucidation of the mechanism of quenching possible. It was found that thioglycollic acid-capped CdS QDs has a shorter life time owing to photo-induced electron transfer, when the oxidized form of the substrate o-phenylenediamine is introduced. Furthermore, on interacting with 3,3-diaminobenzidine a red shift of fluorescence peak was observed. 3,3-diaminobenzidine oxidation polymerization induces an aggregation of thioglycollic acid-capped CdS QDs whose quenching, represented in Figure 18, is proportional to the glucose concentration.

### 4.6. Nonlocal Theories

Nonlocal theories that have been put forward recently have made it very convenient to understand the dynamics of sensors as they experimentally verify the exerted effects over distant systems. They rely mostly on essential features of local uncertainty relations pertaining to physical states. A. Cemal et al. [95] showed pioneering results in implementing localization residuals to account for the interaction of various parts of the system with the material point. The constitutive theory is supported by both classical continuum mechanics and Gibbsian thermodynamics that lead to similar results. The constitutive equations derived also show up in future reports, which give insights into the application of deformation theory to functionally graded plates [96] and infinite-element procedure applied to variational formulations in a group of nonlocal elastic continua [97]. These nonlocal counterparts to traditional principles have proved useful to recover Hu–Washizu principle and Hellinger–Reissner functional.

An equally powerfully sensing technique is offered by strain gradient methods with a high potential to monitor structural integrity. At microscale, such studies are highly valuable in developing a general methodology to provide finite element solution to strain-gradient elasticity problems based on “deformation theory of plasticity”. The developed finite element solution is extended to “mixed” finite element formulation [98] to study well known problems “problem of a plate with a hole” and “a mode-III crack”. A novel modification of nonlocal strain gradient elasticity in the form of size-dependent constitutive formulation has recently been applied to structural modeling of carbon nanotubes [99]. These well-posed mechanical formulations, validated by molecular dynamics simulations, not only serve as a tool in studying the geometrical properties and structural responses. In conjunction, they are exploited beneficially to solve many other problems of nano-mechanics, such as formulation of stress-driven nonlocal integral model and incremental nonlocal elastic equilibrium equations to detect buckling loads of nano-beams [100]. Despite the wide usage of nonlocal theories in the field of elasticity, its application is limited through differential mode in most cases. However, some researchers have adopted the integral mode of this theory to arrive at significant results in nanosystems, such as nanorod, nanoplate, nanotube, and nanoshell, which are reviewed by Mojtaba Shariati et al. [101]. It is evident that nonlocal models find a prominent place in the future and the approach of nonlocal solutions has proved to be a valid efficient theory with wide applications in nanoscale engineering and sensing.

## 5. Regulatory Facet of Nano-Enabled Sensors

On the way of moving from traditional perspective towards the commercialization of innovative recognizing agents, an insight and knowledge of regulatory science bestows a great advantage to the scientific community. With a new approach of regulatory science recognizing the innovations in sensor technology, good practice guidelines can transform the procedural aspects of the research. All sensing devices to be legitimately used are subjected to ordinance with a stringent certification path around the world and, hence, require regulations to ensure accuracy, secured usage, quality, and sharing of data.

Conformity to federal, territorial state, and local regulations is critical, without which the usage of sensors may result in penalties in terms of monetary fines or criminal charges against the user. Michael et al. [102] and Alice et al. [103] have provided a comprehensive review pertaining to current and upcoming regulations of wearable sensors used in clinical and preclinical evaluation. The authors of the former clearly explain the medical device regulations in general, and the latter elaborate the contribution of the European Union, the International Electrotechnical Commission, and the International Organization for Standardization, that validates the design, accuracy, focus, and wearable sensors. A high level of compliance to the regulatory restrictions imposed by statutory bodies have a positive impact on ensuring innovation in designing safe and robust nano-enabled sensors.

Furthermore, an increase in demand for automotive sensors with upraise of governmental regulations is observed. The wireless nanosensors controlled remotely are an integral part of breakdown maintenance, exhaust, engine throttle position, fuel storage, power steering, airbags, brake, and suspension and have transformed the efficiency and safety level in the automotive sector. However, as nano-enabled sensors are a relatively new area of development, these regulations require a consistent updating, compliance with industrial processes, and must undergo licensing agreements. As registering the new compounds used in the sensor is seldom noticed, a detailed evaluation of sensing mechanism and specific regulations are lacking in most areas of nano-enabled sensing and, thus, redefining the existing regulatory framework is becoming increasingly important.

## 6. Safety Concerns in Nano-Enabled Sensors

**Figure 19:**
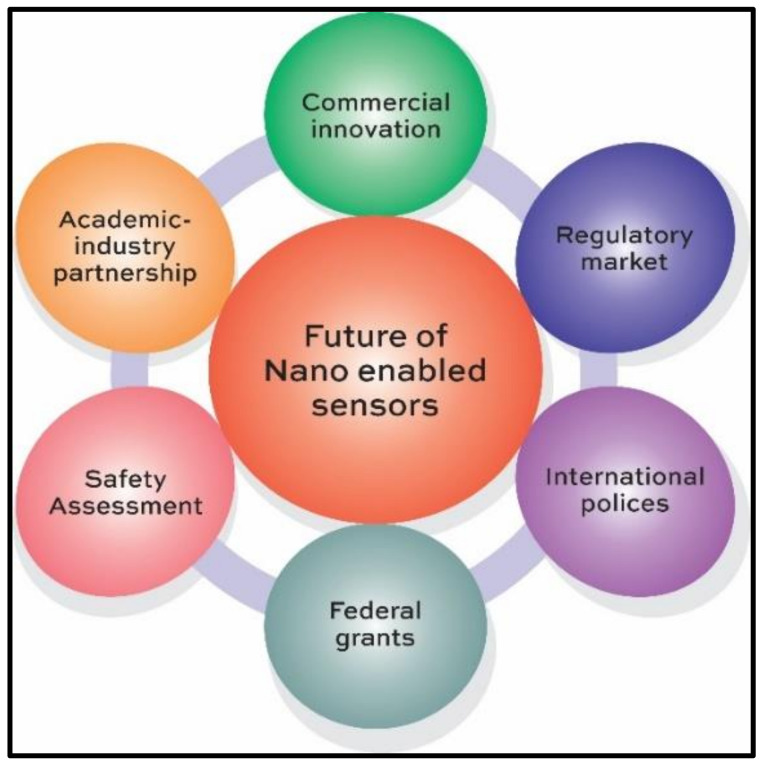
Factors affecting the future development of nano-enabled sensors.

The circular economy ahead for the next decade is predicted to be driven by the trends in sensor-based network systems [104]. As developments over the recent past years have made sensors to find widespread use in the form of an array of devices and play a key role in various fields of technology, it has also made them dependent on numerous financial, political, and scientific efforts for its future expansion, as represented by Figure 19. The advantages and structural problems of nano-aided sensors extends new opportunities to the transforming societies exerting a profound impact on the development of economy.

**Figure 20:**
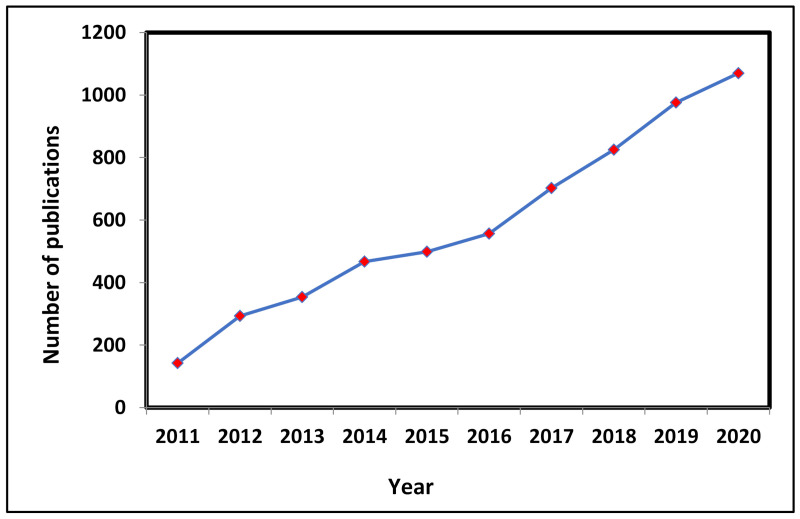
Research publications on nanotechnology-enabled sensors during the last decade.

Despite the much acclaimed potential and consistent innovations in the sensors industry to meet the market’s trends, the intrinsic features of nano-enabled sensors are associated with huge challenges in terms of scientific methodology, handling of nanoparticles, features of the analytes, and environmental soundness. The graph in Figure 20 symbolizes a consistent contribution by the research community towards the growth of nano-enabled research during the last decade [105].

Nevertheless, the development of sensors and implementation of the same for long-term use has always sparked controversy regarding sustainability.

Hence, life cycle analysis, toxicity estimations, and assessment of side effects have become highly crucial for subsequent generations of nano-enabled sensors. The European Union has taken up a huge political task of implementation of sensors and has prescribed the application of precautionary principles of various categories, such as “Margin of safety—Precautionary” to define and limit the parameters within which the no harm to the environment occurs, “Premier available technology—Precautionary” to avail the scientific methodologies with minimum risks, and “Prohibitory- precautionary” to wade the strategies that pose scientific uncertainty.

While these regulatory measures are gaining importance, it brings a great deal of responsibility onto the shoulders of researchers to explore and evaluate the possible ecofriendly processes, and urges the common man to look for alternatives to environmentally harmful actions.

Aleksandra Zielinska et al. [106] have systematically summarized the safety concerns of nanosystems arising from their small size and large surface, which are evaluated by in vitro and in vivo case studies. The authors have a view that a vast majority of nanosystems have minimum invasion on biological processes and intracellular traffic, and an insignificant effect on biological responses in the body due to an easy arrest of these particles by macrophages in the lungs and are often excreted along with the feces.

Unlike in the United States, the European nations, and China, where the initiatives for nanosensors are long established, the regulatory framework of safety measures in lower-middle-income countries needs to be strengthened with new legislative policies. As the international competition is on the rise, a consistent vision of the federal agencies with a focus on investing in private sectors and nanotech research institutes will play a crucial role in fostering the development of nano-enabled sensor technology.

## 7. Unresolved Problems and Future Outlook

Despite the acceptance of nanosensors worldwide for their applications from switching street lights on and off to enabling weather forecasts, the requirement to refine the chemical sensing technology is often overlooked. An article by Andreas et al. [107] provides an outline of advantages and problems associated with miniaturization at the nano scale with current research trends focusing on bio-sensing applications. There are very few sensors for the constant monitoring of important parameters in healthcare and regulating environment.

The terms “Sensors” is often used synonymously with “detectors”, which cannot specifically sense a chemical species and also chemical sensors have a small market share in contrast to sensors working on physical parameters, such as location, air pressure, or temperature. Except for few sensors, such as fluorescence-based oxygen sensors and enzyme-based glucose sensors, which has become a reality, most research is of the academic type and has a long way ahead to be called “ready to use”. Furthermore, the National Materials Advisory Board has identified sensing techniques as a weak link in intelligent processing due to some fundamental concerns regarding the performance of currently available sensors. The real-world constraints are often related to the following:

*High speed sensing*—Registers localized changes and fails to collect the large gradients on the material spanning over long hours of processing.

*Chemical interference*—Simultaneously occurring irrelevant reactions and by-products are sensed.

*Site measurement*—Errors in sensing critical points on the three-dimensional material, especially with changing reaction rates.

Advancements in sensing technology are also limited by the lack of common terminology or descriptors in communicating the needs, performance, and efficiency of sensors. To address this issue, the “Committee on New Sensor Technologies: Materials and Applications” has introduced a few descriptors that correlates sensor application essentials to sensor technology features, and vice versa, thereby helping the research community to identify the potential area for sensors.

It is likely that the progress of sensors would contribute more to society if the following issues are realized:

- Sensors are much more than just a molecule, and, hence, the molecular recognition through mere analytical protocols or “sensing schemes” would not suffice.
- Sensors must be applicable as such to monitor a chemical substance under varying environmental conditions.
- Sensors must be able to operate over different durations, such as few hours through a surgery and a few years as a component in automobile, or a month to monitor glucose in blood of diabetic.
- Sensors must support reversible reactions and respond reversibly towards surrounding oxygen, temperature etc.
- Sensors must not be equated to probes, as probes cannot undergo irreversible reactions while responding to unidirectional signals.

Nevertheless, the new developments in the research of nanotechnology have largely influenced nanosensors such as glucose sensors, gaining a longer lifetime and efficient probes coming up to detect As (II) or Hg (II) ions in ground water; the future of nanosensors is highly optimistic.

Future nano-enabled sensors are predicted to have more specificity, be able to generate more than a trillion data points, and have a higher integrability towards aerial drones and mobile phone networks. As envisioned by many researchers, the nano-equipped sensors will also become self-powered by solar energy or minute fuel cells.

## 8. Our Contribution to This Field

We have synthesized nano MnFeO_4_ [76] using the solution combustion method, which has been used to modify a graphite electrode to detect paracetamol and D-glucose at small concentrations of 1–5 mM along with the ability of decolorizing methylene blue and alizarin red dye. The sensitivity of the material was evaluated through cyclic voltammetry response curves, which intensified with increasing concentrations of analyte. The work was motivated by the previously noted high activity induced five successive decolourization cycles of methylene blue by MnFe_2_O_4_/ZrO_2_ nanocomposite [57]. Exploring the detection capacity of paracetamol and D-glucose was also extended to doping MnFe_2_O_4_ with Ce [77] which resulted in a wider range of responses over 1 to 10 mM. An excellent cyclic voltammetry validation in neutral and NaNO_3_ media proves its efficiency as a candidate for electrochemical sensing.

## 9. Conclusions

While numerous technologies of nano-enabled sensing are emerging with efficiency, compactness, light weight, and huge memory capacity, this is still a new area with opportunities to develop innovative probing mechanisms that can revolutionize sensing solutions with a lesser response time, higher accuracy, and low power consumption. Based on the evidence collected, it is evident that some problems, such as high expense, complex calibration methods, and cumbersome long term data storage loading problems, still persist. Despite these inescapable problems associated with the emerging nano-enabled sensing, the full potential of nano-enabled sensors can only be realized through a reliable technology, predicting the inherent risks, ensuring safety, timely validation, introducing new standard tests, defining the quality levels, characterization of the technology, assessment of the risks associated, evaluating vulnerability of the support systems, and assessing commercial viability.

However, a great potential is as yet untapped and can be accessed through a unified experimental-theoretical approach of research to innovate, deliver, and effectively utilize appropriate sensing methodologies, which is highly crucial in deciding the future outlook and impact on sustainable commercialization of nano-enabled sensing technology.

## Figures and Tables

**Table 1:** A summary of reviews on nano-enabled sensors published in the recent past.

Year	Reference	Title	Journal	Highlights
2021	[108]	Nano–Enabled sensors for detection of arsenic in water.		
2021	[109]	Challenges and potential solutions for Nano sensors intended for use with foods.	Nature nanotechnology	Provides a critical overview of technical, regulatory, political, legal, economic, environmental health and safety, and ethical hurdles associated with sensors in food industry.
2021	[110]	A review on piezo- and pyroelectric responses of flexible nano- and micro patterned polymer surfaces for biomedical sensing and energy harvesting applications.	Nano energy	Reveals nano-structuring of the surface of biocompatible bio sensing widely applied in the field of microfluidic nano-actuated devices, smart drug-delivery systems, and multiferroic systems.
2021	[111]	Review on Carbon Nanomaterials-Based Nano-Mass and Nano-Force Sensors by Theoretical Analysis of Vibration Behavior.	Sensors	Evaluates the developments in nano-mechanical sensors focussing on modeling perspective, continuum mechanical approaches of carbon nanomaterials, symbolic works of CNTs/GSs/carbyne-based nano-mass and nano-force sensors.
2021	[112]	On-site sensing of pesticides using point-of-care biosensors: A review.	Environmental chemistry letters	Overviews latest biosensors developed, which can be utilized for on-site sensing and optical biosensors are at the forefront of technology with advantages such as easy protocols, simple operation, high sensitivity, broad linearity range, and cost-effectiveness.
2021	[113]	Influence of nanotechnology to combat against COVID-19 for global health emergency: A review.	Sensors international	Discusses the development of Nano-enabled sensors towards quick immunization improvement of COVID-19.
2021	[114]	Micro-Nano Processing of Active Layers in Flexible Tactile Sensors via Template Methods: A Review.	Nano-micro-small	Compares the shortcomings and advantages of Sensors via Template Methods to promote the cross-integration of multiple fields and accelerate the development of flexible electronic devices.
2021	[115]	A review on metal- oxide based *p*-*n* and *n* heterostructured nano-materials for gas sensing applications.	Sensors International	Studies the sensors detecting the morphologies of nano rods, nanosheets, nanobelts, nanoribbons, nanowires, nano flowers, spinel, and their market trends.
2020	[116]	Nano-enabled sensing approaches for pathogenic bacterial detection.	Biosensors and Bioelectronics	A comprehensive discussion of the commonly adopted techniques for bacterial identification and a prospective outlook of challenges and solutions is presented.
2019	[117]	Nano-enabled strategies to enhance crop nutrition and protection.	Nature Nanotechnology	Nano-enabled sensing strategies are presented in crop production with a new perspective of profit margin and regulatory aspects in the future agri-business sector.
2019	[118]	Nano-Enabled Technological Interventions for Sustainable Production, Protection, and Storage of Fruit Crops.	Nanoscience for Sustainable Agriculture	Reviews various aspects of nano interventions of Agro nanotechnology.
2020	[119]	Nano-enabled agriculture: from nanoparticles to smart Nano delivery systems.	Environmental Chemistry	Extends a systematic study of sensors in food production and plant nutrition.
2019	[120]	Biosensors for Epilepsy Management: State-of-Art and Future Aspects.	Sensors	Presents highlights on advancements in state-of-art smart nano-enabled bio sensing.
2019	[118]	Nano-Enabled Technological Interventions for Sustainable Production, Protection, and Storage of Fruit Crops.	Nanoscience for sustainable agriculture	Explores the various aspects of nano interventions through nanosensors in agrinanotechnology.

**Table 2:** Detecting ability and negative impact of nanomaterial used in sensors.

Nanomaterial	Size	Enables Detecting	Negative Impact
Microgel	200–400 nm	Water retention	Alters water acquisition
Nano Biopolymer	40–1000 nm	Nutrient absorption	Alters nutrient acquisition
Multiple emulsions	65–500 nm	Concentration of peptides	Influences secondary metabolite production
Filled microgel	370–970 nm	Soil conditions	Impedimentation of seed germination
Microclusters	250–460 nm	Pesticide detection	Oxidation of pesticides

**Table 3:** Comparison of analytical performance of nano-based sensors in biological detection and the food industry.

Nano Material in Sensor	Sensing Methodology	Advantages	Drawbacks	Ref
Hydrogel hybridised carbon nanotube	Metabolism of microbial species causes variation in conductance of nanomaterial	Real time detection possible	The composition of malt extract agar used in the study can vary due to metabolite change.	[29]
Inorganic semiconductor nanoparticles inserted onto membrane	Membrane potential detection via the quantum confined Stark effect	Simultaneous recording of multiple action potential	The membrane insertion may be uneven.	[30]
MoSe2 nano-urchins	Denaturing of target DNA in real life samples of *Escherichia coli*	Stable and sensitive with insignificant interference	Sensing interface degrades over 14 days.	[34]
Prussian blue nanoparticles	H_2_O_2_ sensitivity indirectly quantifies glucose level.	Eco friendly material with high degree of correlation coefficient	Gold precursor may be required to enhance the sensitivity.	[36]
Aptamer embedded magnetic nanoparticles	Fluorescence emission intensity decreases with intensity of E. Coli	Wide linear range and high selectivity towards adulterated pork samples	Binding properties of aptamer to *E. coli* requires a better insight.	[40]
Screen-printed carbon electrode	Cyclic voltammetry and differential pulse voltammetry	Rapid determination, excellent stability, sensitivity, and good reproducibility	Applicable only in the specific dynamic range and detection limit	[48]

## Data Availability

The data described in this article are available from the corresponding author by reasonable request.
